# Supplementary material for: The human amygdala parametrically encodes the intensity of specific facial emotions and their categorical ambiguity
Source: Nat Commun. 2017 Apr 21;8:14821. doi: 10.1038/ncomms14821 (PMC5413952; doi:10.1038/ncomms14821)
Supplement: Supplementary Information — Supplementary Figures, Supplementary Tables, Supplementary Notes, Supplementary Discussion and Supplementary References [file ncomms14821-s1.pdf]

Supplementary Figure 1

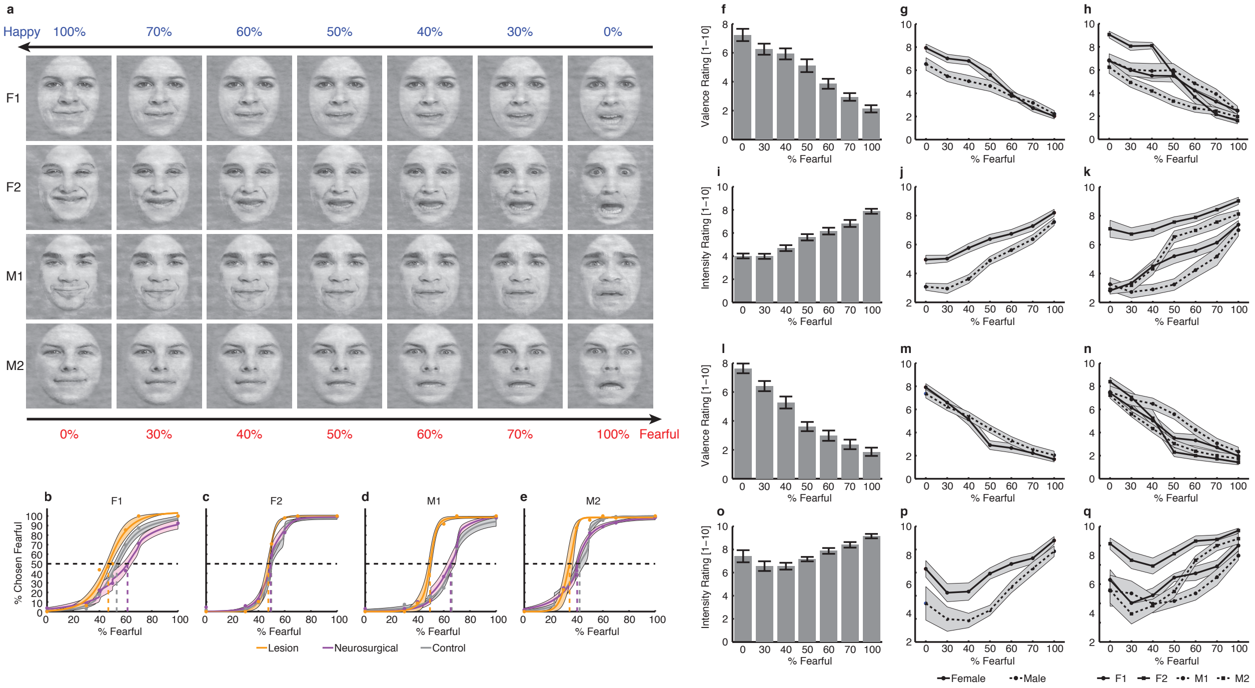

Supplementary Fig. 1. Stimuli and evaluation. **a,** The full set of stimuli used in this study. We selected faces of four identities (2 female) each posing fear and happiness expressions from the STOIC database [1] as anchor faces. We then created 5 levels of fear-happy morphs, ranging from 30% fear/70% happy to 70% fear/30% happy in a step of 10%. **b-e,** Group average of psychometric curves for each facial identity. The psychometric curves show the proportion of trials judged as fearful as a function of morph levels (ranging from 0% fearful (100% happy; on the left) to 100% fearful (0% happy; on the right)). Shaded area denotes ±SEM across subjects/ sessions. **f,l,** Valence ratings across morph levels. **i,o,** Intensity ratings across morph levels. We asked an independent 10 Asian subjects from South China Normal University (similar to the fMRI subjects) and an independent 13 Caucasian subjects from the United States and Germany (similar to the neurosurgical subjects and amygdala patients) to rate the faces. This group of subjects was independent from any of the subject pools that contributed other data to the paper and generated only ratings of the stimuli as shown in this figure. Each face was rated 5 times on a 1 to 10 scale. We asked 'how pleasant is this emotion that the face shows' for valence, with 1 for very unpleasant and 10 for very pleasant. We asked 'how intense is this emotion that the face shows' for intensity, with 1 for very mild/calm and 10 for very intense/excited. Valence ratings

decreased with morph levels whereas intensity ratings increased with morph levels. Error bars denote ±SEM across subjects. **g,j,m,p,** Valence (**g,m**) and intensity (**j,p**) ratings shown separately by subject gender. **h,k,n,q,** Valence (**h,n**) and intensity (**k,q**) ratings shown separately for each identity shown in **a**. Shaded area denotes ±SEM across subjects.

Supplementary Figure 2

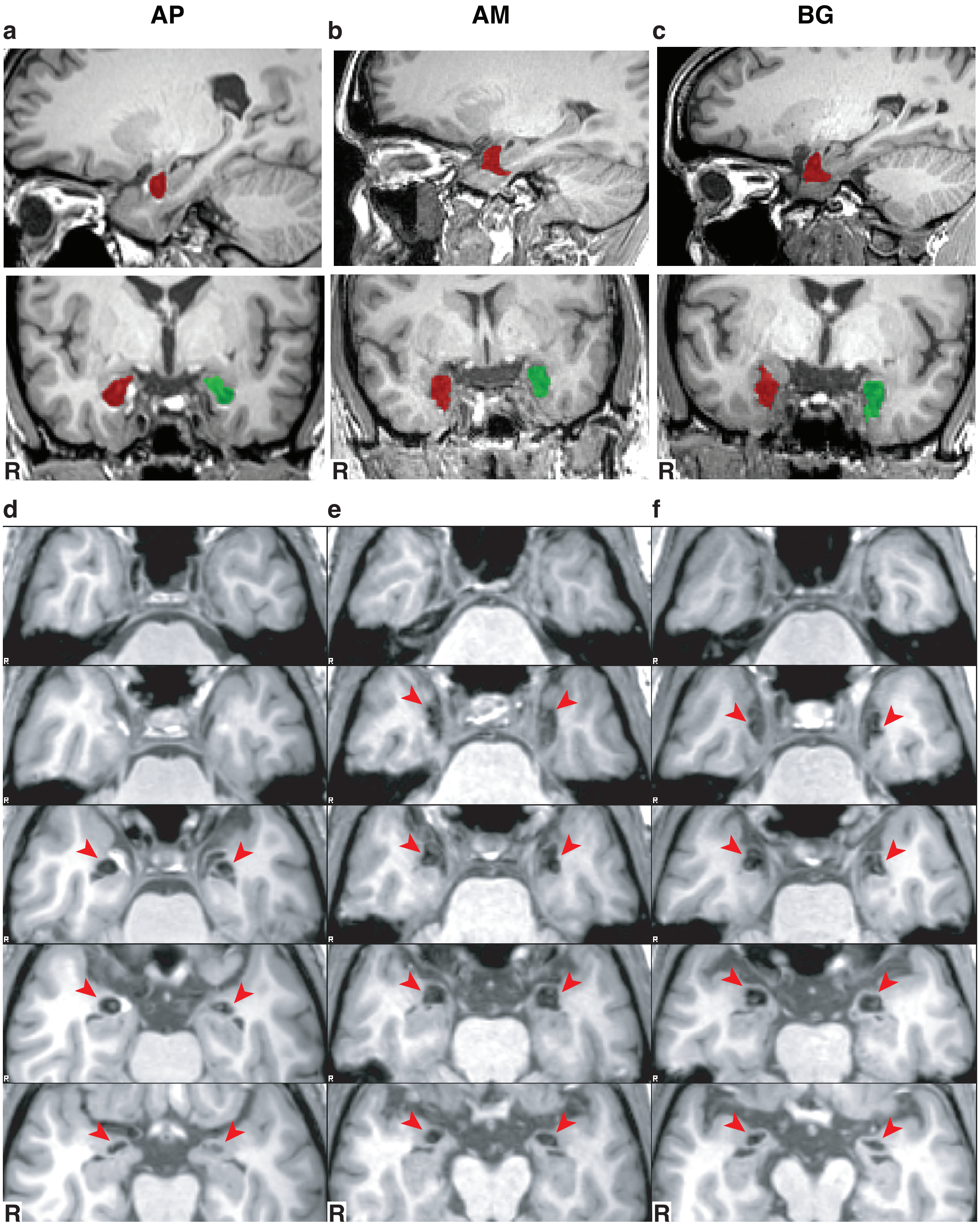

Supplementary Fig. 2. MRI anatomical scans of the amygdala lesions. **a-c,** Sagittal (top row) and coronal (bottom row) sections through T1-weighted structural images of the amygdala lesions for each of the three patients with Urbach-Wiethe disease. Hypo-intense calcified areas are shown in red (right lesion) and green (left lesion). **d-f,** Displayed are high-resolution (0.5-1 mm isotropic) horizontal T1-weighted magnetic resonance imaging sections of the anterior medial temporal lobes with red arrows indexing the focal bilateral amygdala calcification damage. **a,d,** AP; **b,e,** AM; and **c,f,** BG. R: right. In AM and BG, the lesion extended ventrally and caudally from the amygdala into entorhinal cortex. In AP, several hyperintense regions of unknown etiology were observed at the rostral, ventral and medial margins of the lesion, potentially involving the entorhinal cortex.

Supplementary Figure 3

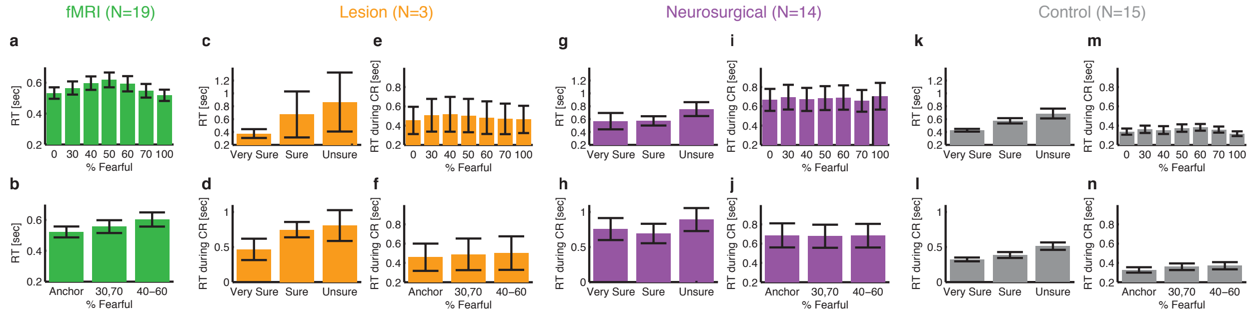

Supplementary Fig. 3. More confidence judgment results. **a,b,** fMRI, **c-f,** lesion, **g-j,** neurosurgical, and **k-n,** control subjects. **a,b,** Though without explicit confidence ratings, fMRI subjects also showed inverted U-shaped reaction times with respect to morph levels, consistent with lesion, neurosurgical and behavioral healthy controls. **b-n,** Subjects judged facial emotions faster when they subsequently indicated higher confidence (**c, g, k**), and they tended to report confidence faster for higher confidence, especially for lesion and control subjects (**d, h, l**). However, subjects did not show difference in reaction times of reporting confidence for different morph levels (**e, i, m**) or ambiguity levels (**f, j, n**). The behavioral patterns of all three subject groups were comparable. Error bars denote one SEM across subjects/sessions.

Supplementary Figure 4

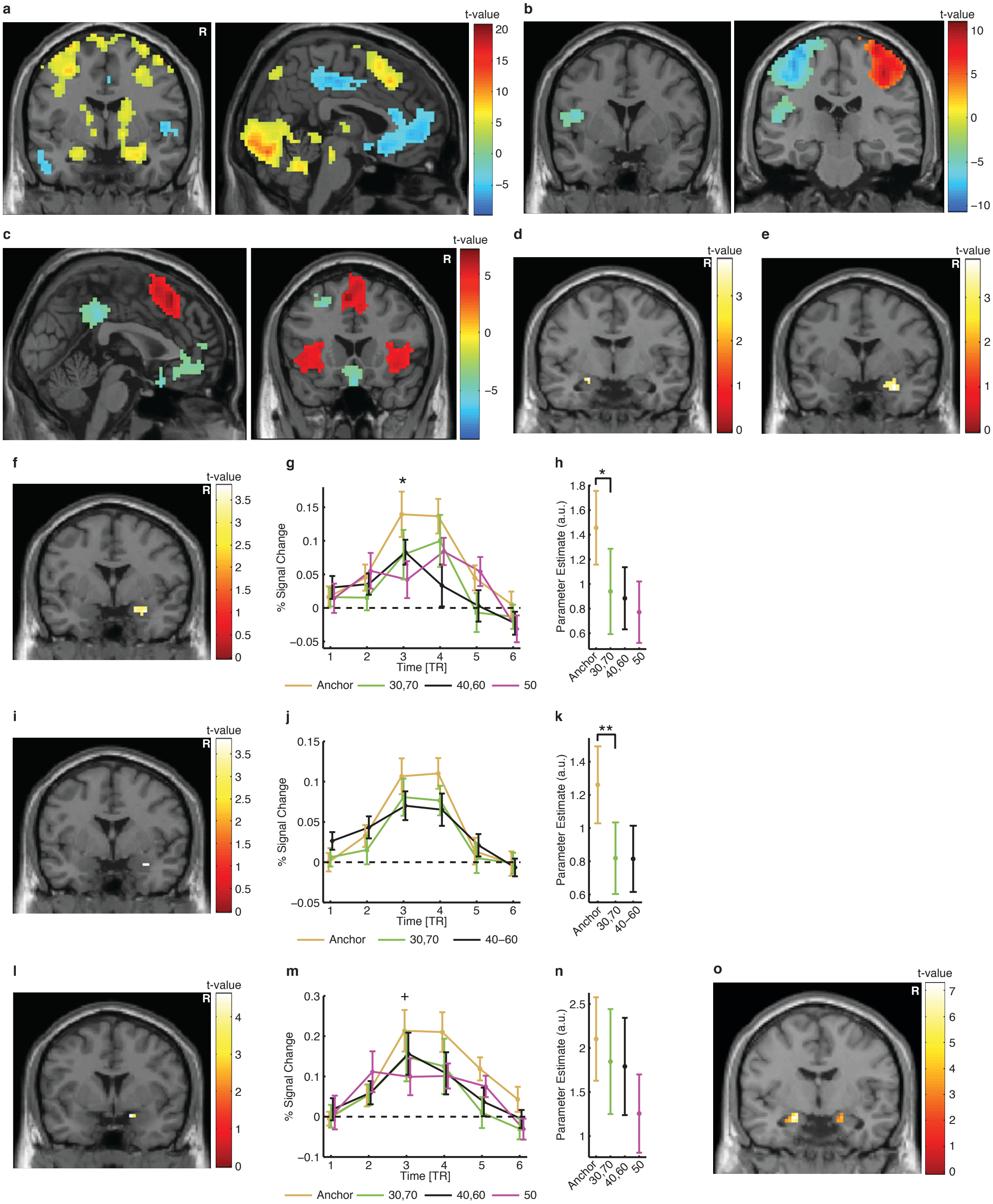

Supplementary Fig. 4. fMRI control analysis. **a,** Localizer task. The face−object condition revealed significant differences in bilateral amygdala as well as the fusiform face area (FFA),

visual cortex, inferior frontal gyrus (IFG), dorsal medial prefrontal cortex (DMPFC), ventral anterior cingulate cortex (ACC), and posterior cingulate cortex (PCC). Hot color represents higher activity in the face condition than in the object condition, whereas blue cold color represents lower activity. Left: coronal view. Right: sagittal view of the right hemisphere. Images are in neurological format with subject left on image left. R: right. **b**, Brain activity modulated by increasing fear levels in the face morph task. Besides the amygdala, motor cortex and left anterior insula were significantly correlated with fear levels. **c**, Brain activity modulated by increasing ambiguity levels. Besides the amygdala, DMPFC/ACC, bilateral anterior insula, ventral ACC, and PCC were significantly correlated with ambiguity levels. Left: sagittal view of the left hemisphere. Right: coronal view. **d**, The left amygdala increased activity with decreasing fear (increasing happy) levels using anatomical amygdala ROI. **e**, The right amygdala increased activity with decreasing ambiguity levels using anatomical amygdala ROI. **f-h,** Regressor with four ambiguity levels. **i-k,** Regressor with three ambiguity levels and modulator of RT. **l-n,** Regressor with four ambiguity levels and modulator of RT. **f,i,l,** Ambiguity levels correlated with BOLD activity in the right amygdala (functional ROI defined by localizer task). Images are in neurological format with subject left on image left. R: right. **g,j,m,** Time course of the BOLD response in the right amygdala (averaged across all voxels in the cluster) in units of TR (TR=2 s) relative to face onset. Error bars denote one SEM across 19 subjects. One-way repeated ANOVA at each TR: *: $P<0.05$, and +: $P<0.1$. **h,k,n,** Parameter estimate (beta values) of the general linear model (GLM) for each ambiguity level (one-way repeated-measure ANOVA: **h,** $P=0.044$; **k,** $P=0.0019$; **n,** $P=0.049$). Error bars denote one SEM across subjects. Asterisks indicate significant difference between conditions using paired two-tailed t-test. *: $P<0.05$, and **: $P<0.01$. **o,** Significantly greater BOLD response in the amygdala for faces vs. fixation. Here we used a functional amygdala ROI defined by the localizer task (see **Methods**).

Supplementary Figure 5

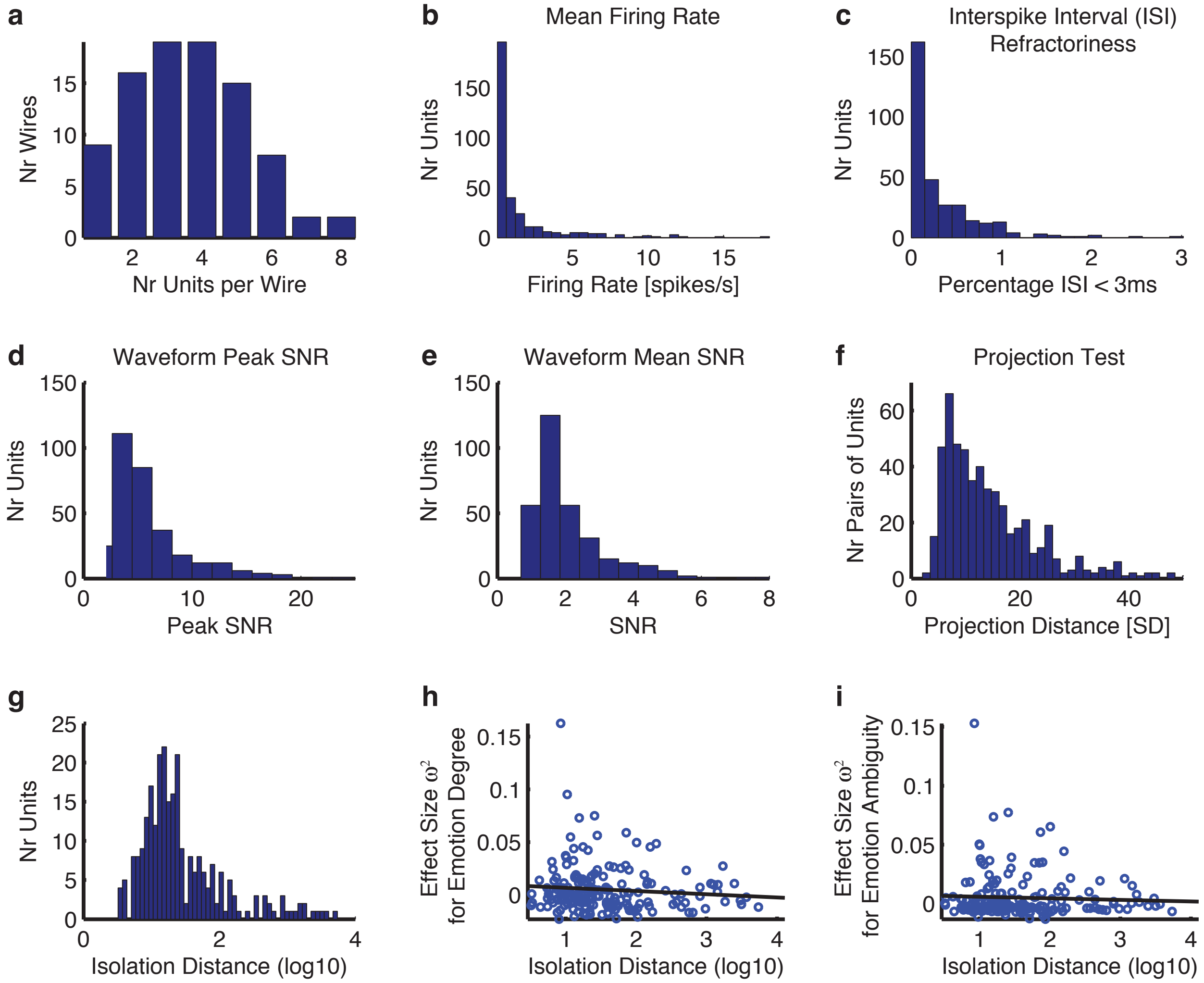

Supplementary Fig. 5. Spike sorting and recording quality assessment. **a,** Histogram of the number of units identified on each active wire (only wires with at least one unit identified are counted). The average yield per wire with at least one unit was 3.63±1.67 (mean±SD). **b,** Histogram of mean firing rates. **c,** Histogram of proportion of inter-spike intervals (ISIs) which are shorter than 3ms. The large majority of clusters had less than 0.5% of such short ISIs. **d,** Histogram of the SNR of the mean waveform peak of each unit. **e,** Histogram of the SNR of the entire waveform of all units. **f,** Pairwise distance between all possible pairs of units on all wires where more than 1 cluster was isolated. Distances are expressed in units of SD after normalizing the data such that the distribution of waveforms around their mean is equal to 1. **g,** Isolation distance of all units for which this metric was defined (n=264, median=18.9). **h,** Absence of

correlation between isolation distance and response strength (as quantified by ω^2) for emotion degree coding (r=−0.094, P=0.21). **i,** Absence of correlation between isolation distance and response strength (as quantified by ω^2) for emotion ambiguity coding (r=−0.047, P=0.53). Each circle represents a neuron (all neurons are shown, regardless whether selected or not). The black line represents the linear fit.

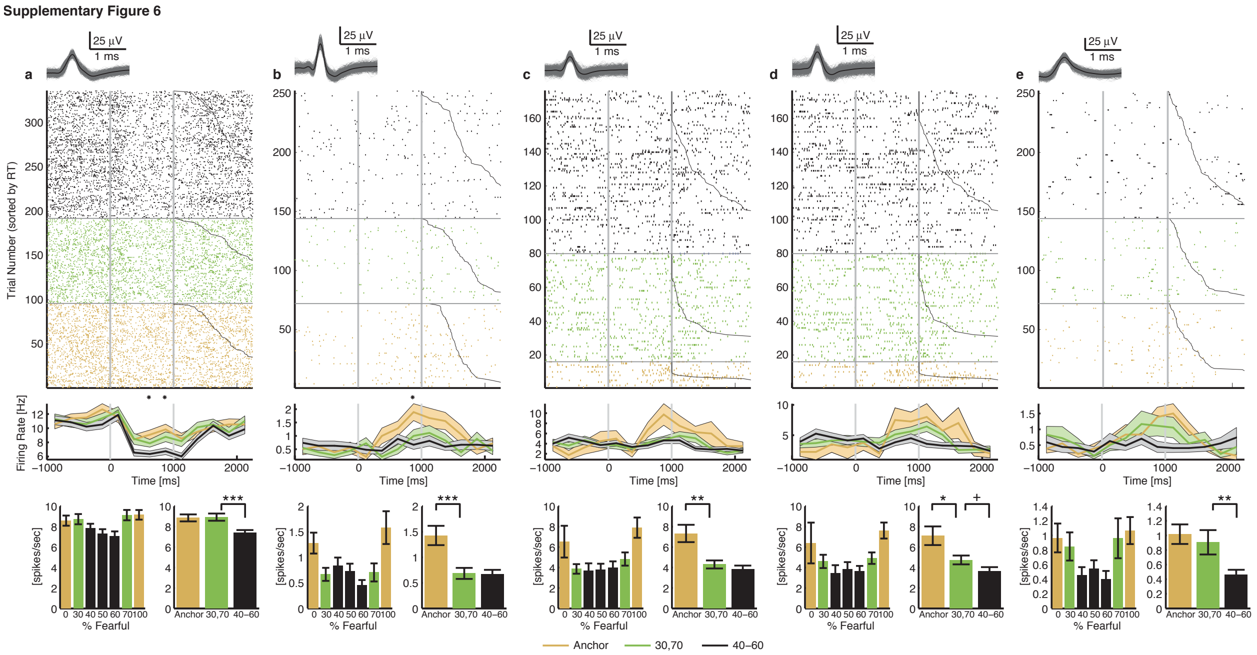

Supplementary Fig. 6. Single-unit examples of ambiguity-coding neurons. Each raster (upper), PSTH (middle) and average firing rate (bottom) is shown with color coding as indicated. Trials are aligned to face stimulus onset (left gray bar, fixed 1s duration). Trials within each stimulus category are sorted according to reaction time (black line). PSTH bin size is 250 ms. Shaded area denotes ±SEM across trials. Asterisk indicates a significant difference between the conditions in that bin (P<0.05, one-way ANOVA, Bonferroni-corrected). Waveforms for each unit are shown at the top of the raster plot. Example neurons show a linearly increasing firing rate for less ambiguous stimulus (linear regression: P<0.05). Bottom left shows the average firing rate for each morph level 250- to 1750-ms post-stimulus-onset. Bottom right shows the average firing rate for each ambiguity level 250- to 1750-ms post-stimulus-onset. Error bars denote ±SEM across trials. Asterisks indicate significant difference between levels of ambiguity using unpaired two-tailed t-test. *: P<0.05, **: P<0.01, ***: P<0.001, and +: P<0.1.

Supplementary Figure 7

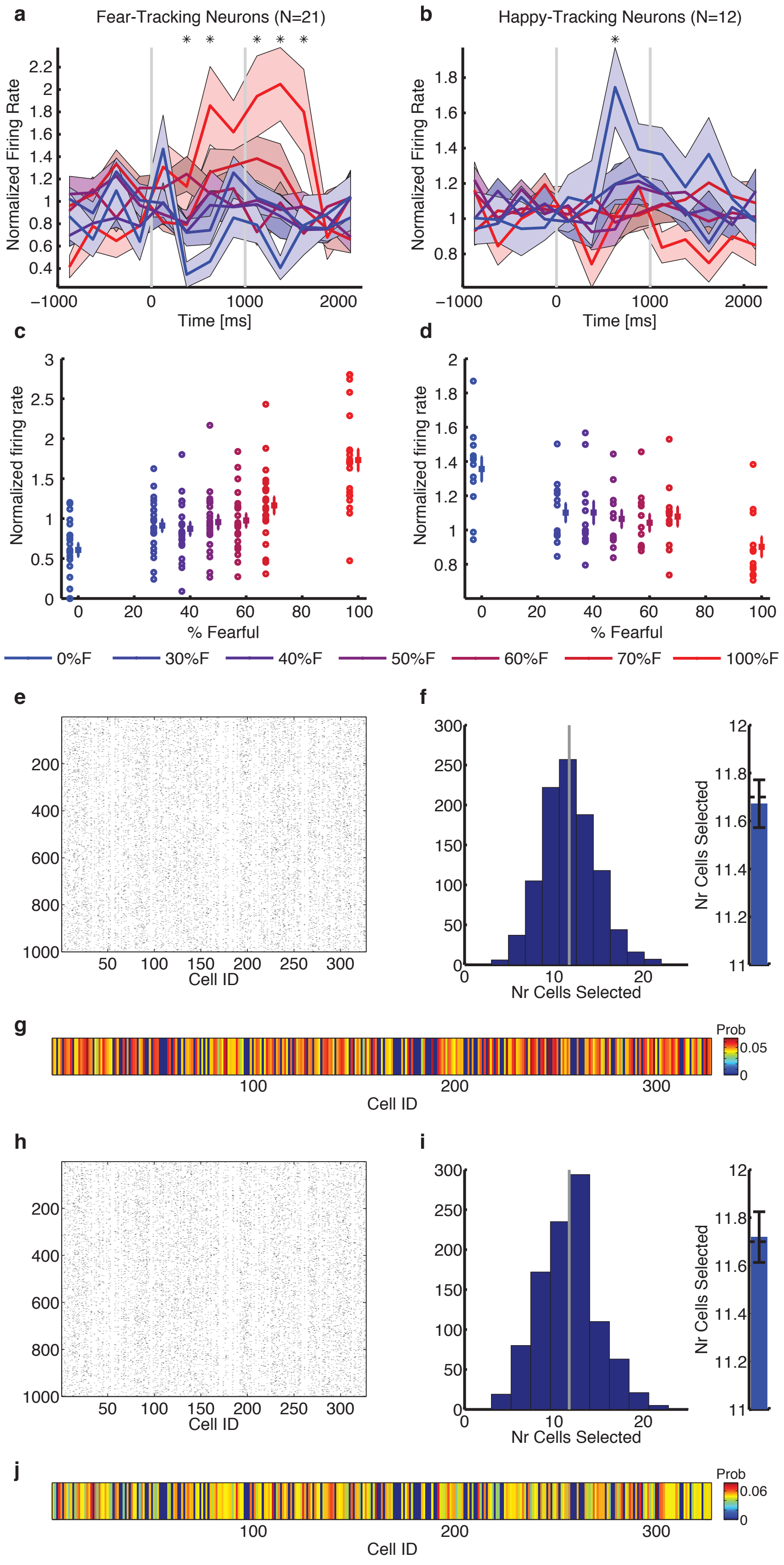

Supplementary Fig. 7. Group and control analysis of emotion tracking and ambiguity coding. **a-b,** Average PSTH of all emotion-tracking neurons. Firing rate of each neuron was normalized separately according to baseline before averaging. **a,** Average PSTH of 21 units that increased their spike rate for fearfulness on faces. **b,** Average PSTH of 12 units that increased their spike rate for happiness on faces. The left gray bar shows face stimulus onset (fixed 1s duration). Bin size is 250 ms. Shaded area denotes ±SEM across units. Asterisk indicates a significant difference between the conditions in that bin ($P<0.05$, one-way ANOVA, Bonferroni-corrected). **c,** Mean normalized firing rate at each morph level for 21 units that increased their spike rate for fearfulness. **d,** Mean normalized firing rate at each morph level for 12 units that increased their spike rate for happiness. Normalized firing rate for each unit (left) and mean±SEM across units (right) are shown at each morph level. **e-j,** Quantification of the permutation test for emotion tracking (**e-g**) and ambiguity coding (**h-j**). **e,h,** Cells selected across runs. Each black dot means a particular cell was selected. Cell selection was evenly distributed across cells and runs in the permutation test, showing no consistency or selection bias. **f,i,** Summary of the number of cells selected. The number of cells selected in the permutation test was near chance (gray bar in the left histogram and black dashed line in the right bar plot). Error bar denotes ±SEM across permutation runs. **g,j,** Summary of the likelihood of each cell being selected. Each cell was equally likely to be selected with the predetermined false discovery rate of 0.05.

Supplementary Figure 8

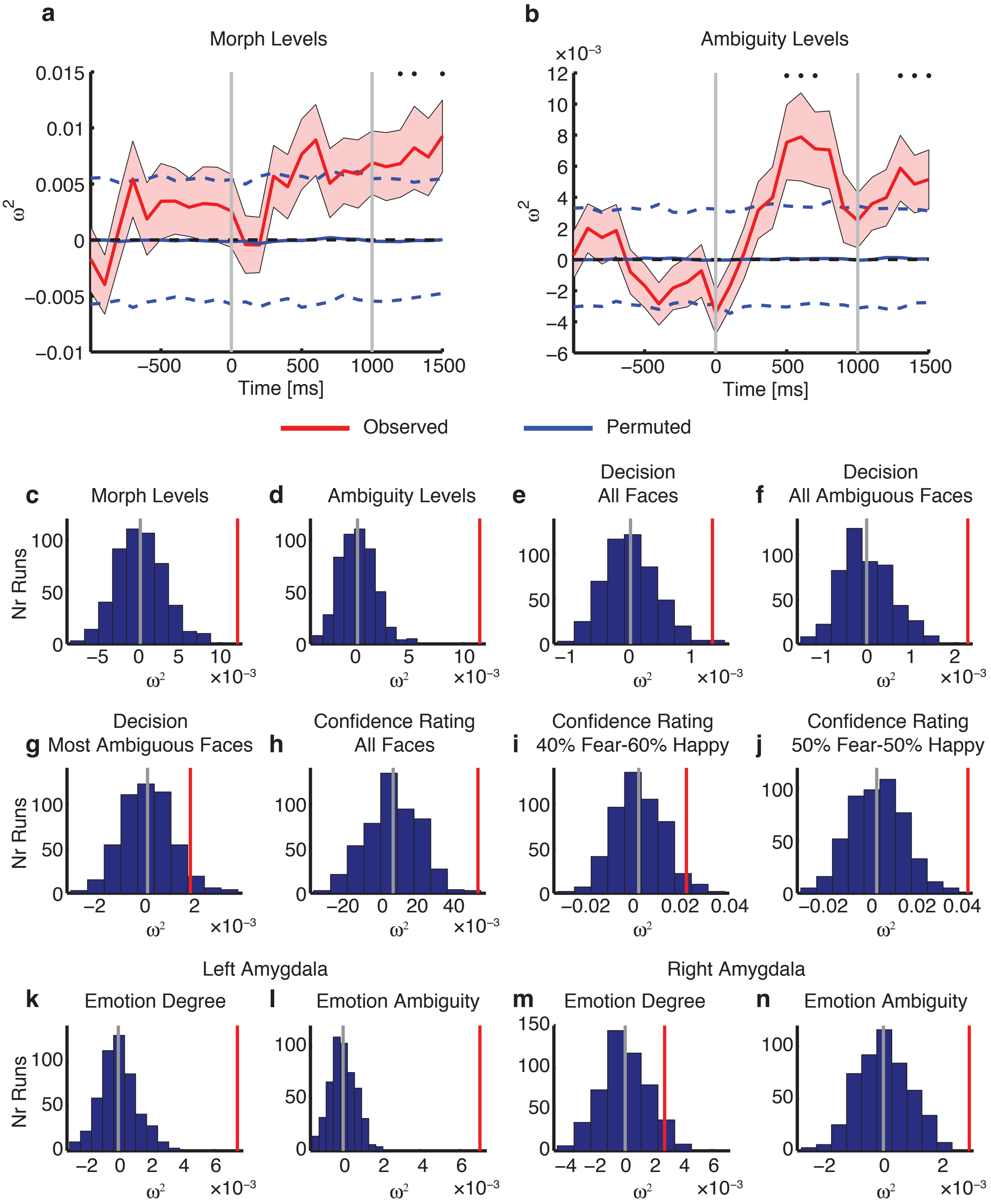

Supplementary Fig. 8. Population analysis with balanced trials in the regression model. Figure legend is the same as **Fig. 5**. **a,b,** Time course of the effect size averaged across all neurons. **a,**

Morph levels. **b,** Ambiguity levels. **c-n,** Summary of the effect size across all runs. Effect size was computed in a 1.5-second window starting 250 ms after stimulus onset (single fixed window, not a moving window) and was averaged across all neurons for each run. Gray and red vertical lines indicate the chance mean effect size and the observed effect size, respectively. **c,** Regression model for morph levels (permutation P<0.001). **d,** Regression model for ambiguity levels (P<0.001). **e-g,** Regression model for decision of emotion (fear or happy) with **e,** all faces (P=0.006), **f,** all ambiguous faces (all morphed faces; P<0.001), and **g,** the most ambiguous faces (40%-60% morph; P=0.054). **h-j,** Regression model for confidence judgment **h,** with all faces (P<0.001), **i,** at the 40% fear/60% happy morph level (P=0.036), and **j,** at the 50% fear/50% happy morph level (P<0.001). **k-n,** Regression model for left (**a,b**) and right (**c,d**) amygdala. Left amygdala neurons as a population encoded both emotion degree (**a,** P<0.001) and ambiguity (**b,** P<0.001). Right amygdala neurons as a population also encoded both emotion degree (**c,** P=0.064) and ambiguity (**d,** P<0.001).

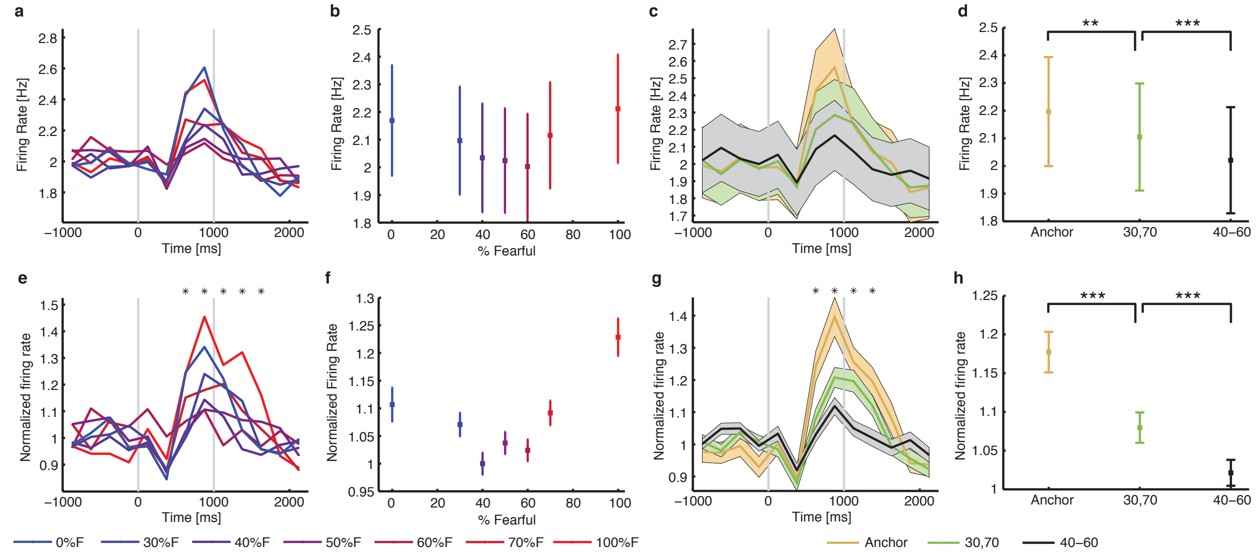

Supplementary Fig. 9. Group PSTH and summary plots for all amygdala neurons. **a-d,** Analysis with raw firing rate. **e-h,** Analysis with normalized firing rate. Firing rate of each neuron was normalized separately according to baseline before averaging. **a,b,e,f,** Analysis at each morph level. **c,d,g,h,** Analysis at each ambiguity level. **a,c,e,g,** Average PSTH across all neurons. Trials are aligned to face stimulus onset (left gray bar, fixed 1s duration). Shaded area denotes ±SEM across units. Asterisk indicates a significant difference between the conditions in that bin ($P<0.05$, one-way ANOVA, Bonferroni-corrected). Bin size is 250 ms. **b,d,f,h,** Mean firing rate at each level. Error bar denotes mean±SEM across units, U-shaped overall response curves (**b,f**) and decreasing overall firing with increasing emotion ambiguity (**d,h**) are evident when averaging across all recorded amygdala neurons. Asterisks indicate significant difference using paired two-tailed t-test. **: $P<0.01$, and ***: $P<0.001$.

Supplementary Table 1. List of patient demographics, pathology, and neuropsychological evaluation.

ID	Age	Sex	Hand	Epilepsy diagnosis	WAIS-III					WMS-R/WMS-III (*)						Rey-Osterrieth		
					PIQ	VIQ	VCI	POI	FSIQ	VPA1	VPA2	LM1	LM2	Vis1	Vis2	copy	IR	DR
C26	56	F	L	Right temporal	107	92	96	109	99	15*	6*	26*	17*	35*	33*	33	16.5	12
C27	45	M	R	Left temporal	79	61	57	80	66	n.a.	n.a.	n.a.	1*	17*	11*	28	4	6
C34	70	M	L	Bilateral temporal	n.a.	n.a.	n.a.	n.a.	n.a.	n.a.	n.a.	13*	2*	11*	0*	1%ile	1%ile	1%ile
H42	29	M	R	Not localized	87	75	78	91	79	16	6	22	14	37	36	36	14.5	14.5
H43	27	F	L	Left temporal	n.a.	n.a.	84	86	n.a.	19	8	18	17	30	24	23.5	14.5	14.5
H44	58	F	L	Right temporal	74	77	72	78	74	12	5	10	3	34	28	29	8	7
H45	41	M	R	Not localized	n.a.	n.a.	114	91	n.a.	14	3	23	22	37	30	35	24.5	26
H47	20	M	L	Right amygdala	105	97	94	111	100	18	8	20	14	36	34	36	23.5	23.5
H48	54	M	R	Left temporal	n.a.	n.a.	76	89	n.a.	0	0	13	8	28	22	33	10.5	16.5

(continued)

ID	Nr Amygdala Neurons								Nucleus	
	Total	>0.2 Hz	Left	Right	Fear	Happy	Unam	Am	Left	Right
C26	32	27	27	0	4	0	10	0	BL	BL
	30	17	17	0	7	0	5	0		
C27	22	15	15	0	1	3	1	0	Ce	BL
	2	2	2	0	0	0	0	0		
C34	41	29	14	15	0	0	5	0	BL	BL
H42	10	9	5	4	1	0	0	0	n.a.	n.a.
H43	40	29	29	0	2	3	1	0	BL	La
	21	17	17	0	1	1	1	0		
	12	8	8	0	0	3	0	0		
H44	23	8	8	0	2	0	0	0	n.a.	n.a.
H45	6	6	0	6	0	0	0	2	n.a.	n.a.
H47	6	6	0	6	0	0	2	0	BL	BL
H48	32	22	5	17	1	2	1	0	BL	BL
	50	39	18	21	2	0	3	1		

Abbreviations: Hand: Dominant handedness; WAIS-III: IQ scores from the Wechsler Adult Intelligence Scale: performance IQ (PIQ), verbal IQ (VIQ), full scale IQ (FSIQ), perceptual organization index (POI), verbal comprehension index (VCI). All WAIS-III scores are on average 100 with a standard deviation of 15 in the normal population (69 and less falls in the clinically abnormal range, 70-79 borderline, 80-89 low average, 90-109 average, 110-119 high average, 120-129 superior, and 130+ very superior). WMS-R and WMS-III are the Wechsler memory scale revised and version 3, respectively. Subtests are verbal paired associates (VPA),

logical memory (LM) and visual reproduction (Vis). 1 and 2 are immediate and delayed, respectively. Scores are raw scores. Scores from the Rey-Osterrieth Complex Figure test are raw scores (except C34 as percentiles) from the subtests copy (visuospatial perception and construction), immediate recall reproduction (IR, additional short-term visual memory demands), and 30-minute delayed recall reproduction (DR, additional longer-term visual memory demands). Tests indicated with n.a. were not performed for clinical reasons.

Patients C26, C27 and H48 performed two sessions and patient H43 performed three sessions (Each row of neurons represent a separate recording session. Each session was recorded on a separate day). >0.2 Hz: neurons that had an overall firing rate greater than 0.2Hz. These neurons were included for further analysis. Left: neurons that were recorded from the left amygdala and had a firing rate greater than 0.2Hz. Right: neurons that were recorded from the right amygdala and had a firing rate greater than 0.2Hz. Fear: emotion-tracking neurons that had higher firing rate for more fearful faces. Happy: emotion-tracking neurons that had higher firing rate for happier faces. Unam: ambiguity-coding neurons that had higher firing rate for unambiguous faces. Am: ambiguity-coding neurons that had higher firing rate for more ambiguous faces. BL: basolateral amygdaloid nucleus. Ce: central amygdaloid nucleus. La: lateral amygdaloid nucleus. Nuclei indicated with n.a. were not mapped due to missing T1 structural images (either pre- or post- operative images).

Supplementary Table 2. Brain areas modulated by fear or ambiguity levels. All values are P<0.001 uncorrected. Asterisk indicates P<0.05 FWE after small volume correction.

	**Brain Region**	**Z-score**	**Peak Coordinate MNI (X Y Z)**	**Volume (voxel)**
Increasing fear	R Motor Cortex (R Precentral Gyrus)	5.96	42 −15 60	1174
	L Fusiform Face Area	4.52	−24 −48 −24	174
	L Supramarginal Gyrus	4.36	−63 −54 21	49
	R Paracentral Lobule	4.31	12 −21 51	47
Decreasing fear	L Anterior Insula	4.60	−45 0 15	221
	L Motor Cortex (L Precentral Gyrus)	5.76	−45 −21 48	1215
	L Amygdala	3.22*	−21 −6 −15	6
	R Fusiform Face Area	5.28	36 −54 −30	378
	L Occipital Cortex/BA 18	4.38	−6 −105 9	136
	L Ventral ACC	4.29	−6 3 51	64
Increasing ambiguity	L Dorsal mPFC/ACC	4.83	−6 15 54	465
	R Dorsal mPFC	3.62	57 18 39	74
	L Inferior Frontal Gyrus/ Anterior Insula	4.64	−30 27 0	215
	R Inferior Frontal Gyrus/ Anterior Insula	4.60	36 21 −6	235
	Caudate Body	4.33	12 9 15	68
Decreasing ambiguity	R Amygdala	3.17*	30 0 −21	17
	L Ventral ACC	4.91	−6 39 −9	454
	L PCC	4.29	−12 −45 42	263
	L Dorsolateral Prefrontal Cortex	4.87	−30 21 45	305

	R Postcentral Gyrus	5.87	57 −24 18	779
	L Inferior Parietal Lobule	5.02	−54 −48 48	819
	Superior Temporal Gyrus	4.50<br>5.50	−63 −18 −30<br>−66 −30 3	157<br>144

Supplementary Notes

Logistic mixed model

We used a logistic mixed model to fit behavioral judgments (fear or happy choice) for all subjects with subject group and fear level as the fixed effects and each subject as the random effect. Statistical significance of the model was computed by likelihood ratio tests of the full model with the fixed effect of subject group or fear level against a null model without the fixed effect of subject group or fear level. We found that in the full model, fear level could predict behavioral judgments with a significant regression coefficient (9.84±0.20 (mean±SEM), 95% CI: [9.44 10.2], $t(333)=49.0$, $P<0.001$), and the full model with the fixed effect of fear level significantly outperformed the null model ($\chi^2(4)=6100.1$, $P<0.001$), suggesting that fear levels could well predict behavioral judgments. Furthermore, in the full model, subject group could not predict behavioral judgments with a significant regression coefficient (0.075±0.064, 95% CI: [−0.051 0.20], $t(333)=1.18$, $P=0.24$), and the full model with the fixed effect of subject group did not significantly outperform the null model ($\chi^2(4)=1.36$, $P=0.24$), suggesting that behavioral judgments were similar among subject groups.

Confidence judgment

We grouped 7 morph levels into 3 ambiguity levels (anchor, 30%/70% morph, 40%-60% morph). Both explicit confidence ratings (one-way repeated-measure ANOVA of ambiguity levels; lesion (**Fig. 1h**): $F(2,4)=3.34$, $P=0.14$, $\eta^2=0.31$; neurosurgical (**Fig. 1l**): $F(2,24)=28.5$, $P=4.59\times10^{-7}$, $\eta^2=0.11$; control (**Fig. 1p**): $F(2,28)=78.6$, $P=3.26\times10^{-12}$, $\eta^2=0.44$; linear regression of the average; lesion: $R^2=0.997$, $P=0.034$; neurosurgical: $R^2=0.965$, $P=0.12$; control: $R^2=0.988$, $P=0.07$) and implicit confidence measures by RT (one-way repeated-measure ANOVA of ambiguity levels; lesion (**Fig. 1i**): $F(2,4)=1.82$, $P=0.27$, $\eta^2=0.075$; neurosurgical (**Fig. 1m**): $F(2,26)=18.4$, $P=1.06\times10^{-5}$, $\eta^2=0.053$; control (**Fig. 1q**): $F(2,28)=26.0$, $P=4.11\times10^{-7}$, $\eta^2=0.14$; linear regression; lesion: $R^2=0.\ 983$, $P=0.084$; neurosurgical: $R^2=0.971$, $P=0.11$; control: $R^2=0.985$, $P=0.077$) demonstrated an linearly increasing pattern with ambiguity levels.

On the other hand, subjects, especially controls, judged facial emotions faster when they subsequently indicated higher confidence (one-way repeated-measure ANOVA of confidence levels; lesion (**Supplementary Fig. 3c**): $F(2,4)=1.22$, $P=0.39$, $\eta^2=0.23$; neurosurgical (**Supplementary Fig. 3g**): $F(2,20)=1.03$, $P=0.37$, $\eta^2=0.047$; control (**Supplementary Fig. 3k**): $F(2,28)=13.9$, $P=6.51\times10^{-5}$, $\eta^2=0.23$; linear regression; lesion: $R^2=0.985$, $P=0.078$; neurosurgical: $R^2=0.763$, $P=0.32$; control: $R^2=0.994$, $P=0.049$). Control subjects also tended to report confidence faster for higher confidence (one-way repeated-measure ANOVA of confidence levels; lesion (**Supplementary Fig. 3d**): $F(2,4)=0.55$, $P=0.62$, $\eta^2=0.089$; neurosurgical (**Supplementary Fig. 3h**): $F(2,20)=0.72$, $P=0.50$, $\eta^2=0.024$; control (**Supplementary Fig. 3l**): $F(2,28)=14.0$, $P=6.16\times10^{-5}$, $\eta^2=0.20$; linear regression; lesion: $R^2=0.875$, $P=0.23$; neurosurgical: $R^2=0.445$, $P=0.53$; control: $R^2=0.963$, $P=0.12$). However, inverted U-shape disappeared for reaction times of reporting confidence (one-way repeated-measure ANOVA of morph levels; lesion (**Supplementary Fig. 3e**): $F(6,12)=2.13$, $P=0.12$, $\eta^2=0.0095$; neurosurgical (**Supplementary Fig. 3i**): $F(6,72)=0.51$, $P=0.80$, $\eta^2=0.0014$; control (**Supplementary Fig. 3m**): $F(6,84)=4.46$, $P=5.73\times10^{-4}$, $\eta^2=0.025$) and we did not observe linearly increasing pattern anymore when grouping for ambiguity levels (one-way repeated-measure ANOVA of ambiguity levels; lesion (**Supplementary Fig. 3f**): $F(2,4)=1.64$, $P=0.30$, $\eta^2=0.0062$; neurosurgical (**Supplementary Fig. 3j**): $F(2,24)=0.062$, $P=0.94$, $\eta^2=7.83\times10^{-5}$; control (**Supplementary Fig. 3n**): $F(2,28)=6.25$, $P=0.0057$, $\eta^2=0.021$; linear regression; lesion: $R^2=0.946$, $P=0.15$; neurosurgical: $R^2=0.151$, $P=0.75$; control: $R^2=0.913$, $P=0.19$).

Fusiform face area (FFA) also tracks emotion degree and ambiguity

We first identified a functional ROI within the FFA sensitive to faces using the face localizer task (left FFA: peak: x=−36, y=−54, z=−18, 305 voxels; right FFA: peak: x=48, y=−48, z=−21, 342 voxels). Within the FFA ROIs, we found a significant increase of activity in the left FFA with increasing level of fear (peak: x=−24, y=−63, z=−15, Z=3.76, 11 voxels, FWE $P<0.05$, SVC) whereas we found a significant increase of activity in the right FFA with increasing level of happiness (peak: x=21, y=−60, z=−12, Z=3.73, 11 voxels, FWE $P<0.05$, SVC). We also found a

significant increase of activity in the right FFA with decreasing level of ambiguity (peak: x=30, y=−51, z=−12, Z=3.63, 6 voxels, FWE $P<0.05$, SVC), however, we did not find any significant increase of FFA activity with increasing level of ambiguity.

Control analysis for emotion-tracking neurons and ambiguity-coding neurons

We carried out several control analyses to confirm the emotion tracking (**Supplementary Fig. 7e,f**). When we randomly assigned morph levels for each trial, we observed chance selection (1000 permutations, mean±SD: 11.67±3.15; two-tailed t-test against the number of chance selection (11.7 neurons): P=0.78). Furthermore, when testing with different time windows after face onset, we could also select similar numbers of neurons. We could also select similar numbers of neurons when using equal number of trials at each morph level. Together, we confirmed that neurons in the amygdala parametrically and linearly track gradual changes of facial emotions along the fear-happy dimension.

Using the same selection (linear regression) as emotion-tracking neurons but with only those trials on which patients classified the face as “fear” or “happy”, respectively, we could select 32 (13.7%; binomial $P<10^{-7}$) or 19 (8.12%; binomial P=0.014) neurons as significant, indicating that emotion tracking was independent of behavioral response. Using correctly judged anchor faces (100% fear or 100% happy without ambiguity), we could select 13 neurons (two-tailed unpaired t-test at $P<0.05$; 5.56%; binomial P=0.28) that differentiated fear and happy emotions. Using three levels of emotion degree (anchor fear, all morphs, and anchor happy), we could select 13 neurons (5.56%; binomial P=0.28; linear regression at $P<0.05$). Furthermore, trial-by-trial correlation of firing rate with fear levels revealed 21 (8.97%; binomial P=0.0036) significant neurons, with 14 neurons showing higher firing rate for fearful faces and 7 neurons showing higher firing rate for happy faces. Interestingly, the number of fear-tracking neurons slightly dominated over the number of happy-tracking neurons (χ^2-test: P=0.027), which was even the case for two individual sessions ($P<0.05$; in the left amygdala).

We carried out a permutation test to confirm the ambiguity coding (**Supplementary Fig. 7h-j**). When we randomly assigned ambiguity levels for each trial, we observed chance selection of 11.72±3.34 (mean±SD) neurons (1000 permutations; two-tailed t-test against the number of chance selection (11.7 neurons): P=0.86), among which 6.13±2.51 neurons showed positive correlations (higher response for anchor faces) and 5.58±2.28 neurons showed negative correlations (higher response for more ambiguous faces), in contrast to our observed data with predominantly positive correlations. Together, we confirmed that the ambiguity-coding neurons that we observed were not due to chance.

To further exclude the possibility that different numbers of repetitions for each stimulus level might have resulted in a greater response to anchor faces because the anchor faces were shown less often (i.e., leading to reduced habituation across presentations and thus weaker adaptation [1]), we separately analyzed the recordings performed in sessions where all stimuli (anchors and morphs) were shown exactly the same number of times (see **Methods**). We found very similar proportions of amygdala neurons encoding emotion ambiguity in this group of patients (14 ambiguity-coding neurons among 102 neurons, 13.7%, binomial $P<10^{-3}$; 11 neurons had the maximal firing rate for anchors and 3 neurons had the maximal firing rate for the most ambiguous faces). This percentage of ambiguity-coding neurons was not significantly different from the entire population (χ^2-test). This shows that our results did not arise simply from different levels of adaptation due to unequal numbers of repetitions.

Model comparisons

Did emotion-tracking neurons differentiate continuously between levels of fear/happy in a face, or might they respond only once a certain threshold level of “fear” was present in a face? To confirm that our linear model was a better fit of our data and that our data could be better described by a linear relationship rather than a step-like thresholded model, we assessed how good this linear model was compared to two more complex models using the Akaike Information Criterion (AIC; see **Methods**): a logistic function and a step function. The better a model

explains the data (with penalty on complexity), the smaller its AIC value. Compared to the logistic function model, 32/33 emotion-selective neurons had smaller AIC values for the linear model (ΔAIC=13.2±11.4 (mean±SD); t-test against 0: $P=1.69\times10^{-7}$). Similarly, compared to a step function model, 30/33 emotion-selective neurons had smaller AIC values for the linear model (ΔAIC=5.47±4.29; $P=2.51\times10^{-8}$). As a control, we also performed the same comparison for a group of neurons selected using a "step function" model, comparing fearful vs. happy trials with anchor faces only. While many fewer neurons were significant (13/234), we again found that most were fit best by the linear model, even though they were selected using a binary contrast: compared to the logistic function model, linear fitting was better for all neurons (13/13, ΔAIC=13.6±8.36; $P=7.41\times10^{-6}$), and compared to the step function model, linear fitting was better for 11/13 neurons (ΔAIC=3.56±2.74; $P=5.34\times10^{-4}$).

Valence and intensity

Could the decreased neural responses to ambiguity be explained by systematic variability in valence and intensity? To test this, we acquired valence and intensity ratings on our stimuli from 23 additional subjects (**Supplementary Fig. 1f-q**). Ten subjects were Eastern Asians (**Supplementary Fig. 1f-k**) and 13 were Western Caucasians (**Supplementary Fig. 1l-q**). As expected, there was a relationship between decreasing valence and increasing intensity as a function of the fearfulness of the morphed faces (**Supplementary Fig. 1f,i,l,o**). This is consistent with the general evaluation of happy and fear in a two-dimensional structure of affect [2]. In addition, this control experiment also demonstrates that the subtle and gradual changes of facial emotions could be resolved by subjects, a result we also found for both genders (**Supplementary Fig. 1g,j,m,p**) and face identities (**Supplementary Fig. 1h,k,n,q**). Crucially, the valence/ intensity ratings did not exhibit the U-shaped pattern that we found for ambiguity-coding signals. In particular, note that stimuli with the smallest and largest values on both valence and intensity were equally ambiguous. We therefore conclude that the ambiguity signal was not driven by valence or intensity dimensions. Notably, Asians (**Supplementary Fig. 1f-k**) and Caucasians (**Supplementary Fig. 1l-q**) demonstrated similar ratings.

In addition, when adding the mean intensity rating from Western Caucasians for each face as a covariate into our regression model, we could still select 28 ambiguity-coding neurons (12.0%; binomial $P=7.36\times10^{-6}$; 5 neurons increased firing rate as a function of ambiguity and 23 neurons decreased firing rate as a function of ambiguity), and these neurons had a similar pattern of response to that shown in **Fig. 4**. Furthermore, using the same selection as the ambiguity-coding neurons, we found that only 10 neurons had a significant trial-by-trial correlation with intensity ratings (4.27%; binomial P=0.63), and only 1 of these 10 neurons was a ambiguity-coding neuron. Again, similar results were derived when excluding the neuron that also encoded emotion intensity. Together, our results suggest that the response of ambiguity-coding neurons could not be explained by emotion intensity.

Emotion degree and ambiguity coding by each facial identity

Subsets of amygdala neurons have been reported in prior studies to be sensitive to facial identities [3], an effect which might interact with emotion and ambiguity coding if present also in our data. To explore this possibility, we selected for neurons selective for facial identity (n=24 neurons, 10.3%, binomial $P=3.25\times10^{-4}$). However, none of these neurons were emotion-tracking neurons and only 8/24 neurons were ambiguity-coding neurons. This selection used all trials; when only using anchor trials, we found that there were 10 neurons (4.27%, binomial P=0.63) selective for facial identity and similarly, none of these neurons were emotion-tracking neurons and only 2 neurons were ambiguity-coding neurons. Second, we separately analyzed each facial identity. With Face Model F1 only (**Supplementary Fig. 1a**), we found 22 emotion-tracking neurons (9.40%, binomial P=0.0017; 16 neurons increased firing rate as a function of fear degree and 6 neurons decreased firing rate as a function of fear degree) and 15 ambiguity coding neurons (6.41%, binomial P=0.13; 13 neurons had the maximal firing rate for anchors and 2 neurons had the maximal firing rate for the most ambiguous faces). With Face Model F2 only, we found 21 emotion-tracking neurons (8.97%, binomial P=0.0036; 11 neurons increased firing rate as a function of fear degree and 10 neurons decreased firing rate as a function of fear degree) and 24 ambiguity coding neurons (10.3%, binomial $P=3.25\times10^{-4}$; 20 neurons had the maximal

firing rate for anchors and 4 neurons had the maximal firing rate for the most ambiguous faces). With Face Model M1 only, we found 31 emotion-tracking neurons (13.3%, binomial $P=2.90\times10^{-7}$; 16 neurons increased firing rate as a function of fear degree and 15 neurons decreased firing rate as a function of fear degree) and 20 ambiguity coding neurons (8.55%, binomial P=0.0074; 15 neurons had the maximal firing rate for anchors and 5 neurons had the maximal firing rate for the most ambiguous faces). With Face Model M2 only, we found 23 emotion-tracking neurons (9.83%, binomial $P=7.57\times10^{-4}$; 9 neurons increased firing rate as a function of fear degree and 14 neurons decreased firing rate as a function of fear degree) and 13 ambiguity coding neurons (5.56%, binomial P=0.28; 10 neurons had the maximal firing rate for anchors and 3 neurons had the maximal firing rate for the most ambiguous faces). Our results thus show that although different facial identities had different strengths for selections, emotion-tracking and ambiguity-coding neurons could be selected independently for each identity, and thus encoding of emotion and ambiguity was not driven by facial identity. Note that fewer neurons were selected due to fewer trials and thus reduced statistical power.

Decision coding

First, we found that 20 neurons (8.55%; binomial P=0.0074) differentiated fear and happy decisions using all trials (13 increased and 7 decreased with fear response; unpaired t-test), but only 3 of these neurons were also emotion-tracking neurons and the overlap between these neurons and emotion-tracking neurons was not significant (χ^2-test: P=0.91), suggesting that it was an independent population of neurons compared with emotion-tracking neurons. Breaking down for morph levels, there were 11 neurons (4.70%) showing higher mean firing for fear judgment at all levels whereas there were 9 neurons (3.85%) showing higher mean firing for happy judgment at all levels. At the most ambiguous morph level (50% fear / 50% happy), 15 neurons (6.41%; binomial P=0.13) could differentiate fear vs. happy response, showing that given identical stimulus, neurons could encode subjective judgment, consistent with our previous finding [4]. Similarly, at the most ambiguous 3 morph levels (40% fear / 60% happy to 60% fear / 40% happy), 15 neurons could differentiate fear vs. happy response. Interestingly, 28 neurons

(12.0%, binomial $P=7.36\times10^{-6}$) had a higher firing rate for fear response given fearful faces while a higher firing rate for happy response given happy faces, whereas 5 neurons (2.14%) showed an opposite trend. Such interaction between stimulus and response indicates that these neurons track how much decisions match with stimulus.

Response aligned to button presses and confidence ratings

To provide an initial analysis that might partly separate perceptual and decision processes, we also aligned trials to button presses and quantified the response of each neuron based on the number of spikes in preparation to button press (1s window, starting 1000 ms before button press). We first found that 16 neurons (6.84%; binomial P=0.080) differentiated fear and happy decisions (11 increased and 5 decreased with fear response; unpaired t-test). We could still select 26 emotion-tracking neurons (11.1%, binomial $P=5.29\times10^{-5}$; 14 neurons increased firing rate with fear levels and 12 neurons decreased firing rate with fear levels) and 31 ambiguity-coding neurons (13.3%, binomial $P=2.90\times10^{-7}$; all neurons decreased firing rate with ambiguity levels), suggesting that the representations of emotion degree and emotion ambiguity were still a robust finding, even using this later time window for analysis.

In another analysis of a different temporal window, we aligned response to confidence ratings and quantified the response of each neuron based on the number of spikes in response to confidence rating (1s window, starting at confidence rating onset). Since we omitted confidence rating for Patient C34, all neurons from this patient were excluded. In the remaining 205 neurons, we found 31 emotion-tracking neurons (15.1%, binomial $P=1.25\times10^{-8}$; 15 neurons increased firing rate with fear levels and 16 neurons decreased firing rate with fear levels), 12 ambiguity-coding neurons (5.85%, binomial P=0.23; 9 neurons increased firing rate with ambiguity levels and 3 neurons decreased firing rate with ambiguity levels), as well as 16 neurons (7.80%; binomial P=0.029) that differentiated fear vs. happy decisions (9 increased and 7 decreased with fear response). Since Patient C26 only rated ‘Very Sure’ as confidence across all trials, we further excluded all neurons from this patient (two sessions), resulting in a total 161

neurons remaining for the analysis of confidence. We found that 28 neurons (17.4%, binomial $P=2.13\times10^{-9}$) could differentiate levels of confidence (one-way ANOVA) and 24 neurons (14.9%, binomial $P=5.11\times10^{-7}$) significantly correlated with levels of confidence (trial-by-trial correlation). However, only 2 neurons selected by ANOVA and 1 neuron selected by correlation were emotion-tracking neurons, only 3 neurons selected by ANOVA and 3 neurons selected by correlation were ambiguity-coding neurons, and only 1 neuron selected by ANOVA and 2 neurons selected by correlation also encoded decisions. These results suggest that emotion-tracking and ambiguity-coding representations are no longer evident at this later time window.

Supplementary Discussion

Neuroimaging studies in humans have identified brain areas that co-vary with some parameters of faces in a continuous manner. In particular, using faces with varying levels of trustworthiness, it has been shown that regions in the amygdala track both how untrustworthy a face appeared (i.e., negative-linear responses) and the overall strength of a face's trustworthiness signal (i.e., nonlinear responses), despite faces not being subjectively perceived [5]. In our present study, we found similar parametric effects, not only with neuroimaging but also in direct electrophysiological responses.

Although most studies find activation within the amygdala that is highest for fearful faces [6-8], there are also studies showing that the amygdala responds to neutral or happy faces [9] as well as to some extent all facial expressions [10]. Such general coding of facial expressions is also evident at the single-neuron level [11-14]. Notably, even using the same faces, amygdala BOLD response increases for fearful faces vs. happy faces when using a face mask whereas it decreases for fearful faces vs. happy faces when using a pattern mask [15]. Therefore, the sign of the amygdala's BOLD response to facial emotions may largely depend on the task. In the present study, we found both fear-tracking and happy-tracking neurons. There were overall more fear-tracking neurons than happy-tracking neurons (21 vs. 12; χ^2-test: n.s.), whereas we found a greater BOLD signal for happy faces in the left amygdala. However, these findings are actually not discrepant, since 11 out of 12 neurons showing increasing firing rate with the degree of happiness were in the left amygdala.

The most ambiguous faces are in the middle of a face continuum whereas anchor faces are at the extremes of the continuum. Thus, another possible explanation of the amygdala responses to emotion ambiguity that we observed could be that the amygdala encodes the absolute distance from the average face, consistent with a previous finding that the amygdala has a stronger response to the extremes of the dimensions than to faces near the average face [16]. Furthermore, adaptation studies seek a graded recovery from neural adaptation with ever greater dissimilarity between pairs of stimuli [1]. In the present study, anchor faces are more distinctive compared to ambiguous faces and are thus less subject to adaptation from perceptual neighbors. Therefore,

more pronounced adaptation for stimuli in the middle than at the extremes of the face continuum could in principle be one mechanism that explains the amygdala's response to ambiguity we found in our study. However, we used a sufficient number of distinct stimuli, and their order was completely randomized, making face adaptation not likely in our protocol. Moreover, adaptation to facial emotion was likely to be lower when facial identity also changed on a trial-by-trial basis as in the present study.

In this study, we showed that two closely related variables with meta-information about the decision itself (fear/happy) are represented in the amygdala, one based on objective discriminability of the stimuli, and the second based on the subjective judgment of their discriminability: ambiguity and confidence. This is interesting, because a judgment of confidence is thought to be a direct consequence of an assessment of uncertainty [17]. The mechanisms by which such confidence judgments are made, however, remain poorly understood. It has been suggested that confidence judgments rely on a modified "race to threshold" approach, which relies on integrating the evidence for and against a hypothesis separately. The difference between the two quantities of integrated evidence is proportional to the subjective confidence in the decision [17]. In a recognition memory task, we have recently shown that the activity of a specific subset of human amygdala neurons is compatible with this model: the stronger the integrated difference between a familiarity and a novelty signal carried by individual neurons, the larger the subjective confidence [18]. Together, this raises the important question of whether the amygdala provides a general ambiguity signal that provides the underlying information necessary to judge the confidence in decisions about internal states in general. This important hypothesis remains to be investigated by comparing the activity of the same neurons in several different decision-making tasks.

Many neurons in the macaque amygdala change their firing rate in response to rewards or stimuli predicting the later delivery of rewards or punishments. For example, amygdala neurons differentiate between cues that predict delivery of positively-or negatively valued rewards [19] and they respond to reinforcements that are unexpected [20]. Reward-related coding of uncertainty has also been identified in macaque midbrain dopamine [21] and septal neurons [22], which signal after

cues that predict unreliable rather than reliable rewards [23]. In these studies, individual visual stimuli are associated with the probability of obtaining reward after extensive conditioning. In contrast, in our task no trial-by-trial feedback or reward is delivered, no training on the ambiguity associated with each stimulus is provided and the neurons we identified have a stimulus-evoked response unrelated to reward delivery or expectation. We thus here show a reward- and reward-value independent decreasing response to ambiguity (increasing response to certainty) in the amygdala. Human single-neuron recordings are uniquely suited to test this hypothesis, because no training before the recording is required and patients are able to perform the task without rewards and punishments to incentivize correct performance.

Supplementary References

1 Kahn, D. A. & Aguirre, G. K. Confounding of norm-based and adaptation effects in brain responses. *NeuroImage* **60**, 2294-2299 (2012).

2 Russell, J. A. A circumplex model of affect. *Journal of Personality and Social Psychology* **39**, 1161-1178 (1980).

3 Mormann, F. *et al.* Neurons in the human amygdala encode face identity, but not gaze direction. *Nat Neurosci* **18**, 1568-1570 (2015).

4 Wang, S. *et al.* Neurons in the human amygdala selective for perceived emotion. *Proceedings of the National Academy of Sciences* **111**, E3110-E3119 (2014).

5 Freeman, J. B., Stolier, R. M., Ingbretsen, Z. A. & Hehman, E. A. Amygdala Responsivity to High-Level Social Information from Unseen Faces. *The Journal of Neuroscience* **34**, 10573-10581 (2014).

6 Morris, J. S. *et al.* A differential neural response in the human amygdala to fearful and happy facial expressions. *Nature* **383**, 812-815 (1996).

7 Phillips, M. L. *et al.* Neural responses to facial and vocal expressions of fear and disgust. *Proceedings of the Royal Society of London B: Biological Sciences* **265**, 1809-1817 (1998).

8 Whalen, P. J. *et al.* Human Amygdala Responsivity to Masked Fearful Eye Whites. *Science* **306**, 2061 (2004).

9 Mende-Siedlecki, P., Verosky, S. C., Turk-Browne, N. B. & Todorov, A. Robust Selectivity for Faces in the Human Amygdala in the Absence of Expressions. *Journal of Cognitive Neuroscience* **25**, 2086-2106 (2013).

10 Fitzgerald, D. A., Angstadt, M., Jelsone, L. M., Nathan, P. J. & Phan, K. L. Beyond threat: Amygdala reactivity across multiple expressions of facial affect. *NeuroImage* **30**, 1441-1448 (2006).

11 Fried, I., MacDonald, K. A. & Wilson, C. L. Single Neuron Activity in Human Hippocampus and Amygdala during Recognition of Faces and Objects. *Neuron* **18**, 753-765 (1997).

12 Viskontas, I. V., Quiroga, R. Q. & Fried, I. Human medial temporal lobe neurons respond preferentially to personally relevant images. *Proceedings of the National Academy of Sciences* **106**, 21329-21334 (2009).

13 Rutishauser, U. *et al.* Single-Unit Responses Selective for Whole Faces in the Human Amygdala. *Current biology* **21**, 1654-1660 (2011).

14 Quian Quiroga, R., Kraskov, A., Mormann, F., Fried, I. & Koch, C. Single-Cell Responses to Face Adaptation in the Human Medial Temporal Lobe. *Neuron* **84**, 363-369 (2014).

15 Kim, M. J. *et al.* Behind the mask: the influence of mask-type on amygdala response to fearful faces. *Social Cognitive and Affective Neuroscience* **5**, 363-368 (2010).

16 Said, C. P., Dotsch, R. & Todorov, A. The amygdala and FFA track both social and non-social face dimensions. *Neuropsychologia* **48**, 3596-3605 (2010).

17 Kepecs, A. & Mainen, Z. F. A computational framework for the study of confidence in humans and animals. *Philosophical Transactions of the Royal Society of London B: Biological Sciences* **367**, 1322-1337 (2012).
18 Rutishauser, U. *et al.* Representation of retrieval confidence by single neurons in the human medial temporal lobe. *Nat Neurosci* **18**, 1041-1050 (2015).
19 Paton, J. J., Belova, M. A., Morrison, S. E. & Salzman, C. D. The primate amygdala represents the positive and negative value of visual stimuli during learning. *Nature* **439**, 865-870 (2006).
20 Belova, M. A., Paton, J. J., Morrison, S. E. & Salzman, C. D. Expectation modulates neural responses to pleasant and aversive stimuli in primate amygdala. *Neuron* **55**, 970-984 (2007).
21 Fiorillo, C. D., Tobler, P. N. & Schultz, W. Discrete Coding of Reward Probability and Uncertainty by Dopamine Neurons. *Science* **299**, 1898-1902 (2003).
22 Monosov, I. E. & Hikosaka, O. Selective and graded coding of reward uncertainty by neurons in the primate anterodorsal septal region. *Nat Neurosci* **16**, 756-762 (2013).
23 Tobler, P. N., O'Doherty, J. P., Dolan, R. J. & Schultz, W. Reward value coding distinct from risk attitude-related uncertainty coding in human reward systems. *J Neurophysiol* **97**, 1621-1632 (2007).
